# Evidence from Drosophila Supports Higher Duplicability of Faster Evolving Genes

**DOI:** 10.1093/gbe/evac003

**Published:** 2022-01-09

**Authors:** Zoe Vance, Lukasz Niezabitowski, Laurence D Hurst, Aoife McLysaght

**Affiliations:** Smurfit Institute of Genetics, Trinity College Dublin, Ireland; Smurfit Institute of Genetics, Trinity College Dublin, Ireland; Department of Biology and Biochemistry, University of Bath, United Kingdom; Smurfit Institute of Genetics, Trinity College Dublin, Ireland

**Keywords:** gene duplication, evolutionary rates, duplicability, Drosophila

## Abstract

The faster rate of evolution of duplicated genes relative to singletons has been well documented in multiple lineages. This observation has generally been attributed to a presumed release from constraint following creation of a redundant, duplicate copy. However, it is not obvious that the relationship operates in this direction. An alternative possibility—that the faster rate of evolution predates the duplication event and the observed differences result from a higher propensity to duplicate in fast-evolving genes—has been tested in primates and in insects. However, these studies arrived at different conclusions and clarity is needed on whether these contrasting results relate to differences in methodology or legitimate biological differences between the lineages selected. Here, we test whether duplicable genes are faster evolving independent of duplication in the *Drosophila* lineage and find that our results support the conclusion that faster evolving genes are more likely to duplicate, in agreement with previous work in primates. Our findings indicate that this characteristic of gene duplication is not restricted to a single lineage and has broad implications for the interpretation of the impact of gene duplication. We identify a subset of “singletons” which defy the general trends and appear to be faster evolving. Further investigation implicates homology detection failure and suggests that these may be duplicable genes with unidentifiable paralogs.

Significance Understanding the relationship between gene duplication and evolutionary rates is important for interpretation of the evolutionary consequences of duplication. The classic model is that redundancy relaxes functional constraints and thus permits accelerated evolution. However, this view has recently been challenged by the observation that duplicable genes in primates are faster evolving in the first place. Here, we consolidate that emerging view by showing that this extends to *Drosophila* species, suggesting that it is a general trend.

## Introduction

Gene duplication is an important process in biological innovation with major roles in the evolution of genome structure and content. Duplication may occur by several mechanisms broadly grouped into small-scale duplication (SSD; which includes tandem duplication and retrocopying) and whole-genome duplication (WGD). Genes which successfully duplicate by different mechanisms differ significantly from both singletons and each other in notable ways (Hakes et al. 2007; Amoutzias et al. 2010; Jiang et al. 2013; Banerjee et al. 2017; Qiao et al. 2018). One difference which is frequently seen is that SSDs are less constrained and more dispensable than either WGDs or singletons (Makino et al. 2009).

This lower level of constraint in SSDs is consistently observed as a higher rate of evolution compared with other genes (He and Zhang 2006; Qiao et al. 2018; Defoort et al. 2019). This feature of duplicated genes is of particular interest as it has frequently been used to form hypotheses on the processes of duplicate retention. Specifically, the higher rate of evolution in duplicates has contributed to the idea that duplicates are redundant at the point of creation and thus can accumulate changes free from evolutionary constraint (Lynch and Conery 2000; Jordan et al. 2004; Pegueroles et al. 2013). Knowledge of the true relationship between gene duplication and evolutionary rate has important implications for our understanding of duplicate retention and the role of gene duplication in evolutionary processes. Whereas one model implicates a fixation bias in favor of less constrained, fast-evolving genes (He and Zhang 2006; Woods et al. 2013), the other views these properties as a consequence of duplication and perhaps sometimes instrumental in their successful long-term retention due to sequence and functional divergence of the paralogs, as in subfunctionalization (Force et al. 1999; Des Marais and Rausher 2008) or neofunctionalization (Chen et al. 1997) models.

Despite the faster rate of evolution being one of the most reliably observed features of duplicated genes across lineages, and its relevance to our present understanding of duplicate evolution, the relationship between evolutionary rate and duplicability has not been fully resolved. Does duplication release constraint through redundancy and cause faster rates; or is a faster rate of evolution correlated with a higher chance of successful duplication? Put another way, do SSDs show low constraint because they have additional copies, or are more dispensable genes more likely to be successfully duplicated in the first place?

Previous work on the topic of differentiating these two possibilities has focused on rate measurement in outgroups to approximate the ancestral rate of evolution while assessing duplicability in the ingroups, thereby uncoupling the rate estimation from the duplication event (Davis and Petrov 2004; O’Toole et al. 2018). Although the methods are similar, these two studies examine different lineages, and estimate evolutionary parameters over vastly different periods of time. O’Toole et al. (2018) use closely related primate outgroups to approximate the ancestral rate prior to duplication and find that genes which have duplicated within the apes have higher ancestral rates than those which have not. Similarly, Davis and Petrov (2004) use outgroups to measure the evolutionary rate independently of the species in which duplication has taken place, but they concluded that duplicable genes are more conserved. It is worth noting the large evolutionary distance between the outgroup species used in this study (*Drosophila*  *melanogaster* and *Anopheles*  *gambiae*) and the species where duplicate or singleton status was defined (namely *Saccharomyces*  *cerevisiae* and *Caenorhabditis*  *elegans*).

There are two possible explanations for this contradiction in results. First, there may be genuine biological differences between the lineages which in turn affect the processes governing duplicate retention. Generation time and population size are vastly different when comparing primates (generation times of decades and effective populations in the thousands) to organisms such as yeast (generation time of hours, effective population in the millions). Both of these features have relationships to selection and mutation rate, which may impact on gene duplication and retention as well as evolutionary rates. The history of WGD also differs across the organisms considered in previous studies: whereas primates and *S. cerevisiae* both show evidence of ancient polyploidy (Kellis et al. 2004; Dehal and Boore 2005; Nakatani et al. 2007), there are no WGD events proposed to have occurred ancestral to *D. melanogaster*, *A. gambiae*, or *C. elegans*. Significant differences in the relationship between evolutionary rate and duplication have been observed between duplicates arising from differing mechanisms (Hakes et al. 2007; Makino et al. 2009). However, in both cases, the duplication events examined are not generated by WGD (O’Toole et al. 2018, only consider primate-specific duplicates) so this difference is unlikely to be relevant.

Second, it may be the case that the large differences in divergence time of the outgroups used by the two studies has impacted on the genes included in the analysis. Genes with recognizable orthologs between insects and yeasts probably represent a more conserved group than genes with recognizable orthologs within the primate lineage. Additionally, it is known that genes which are duplicable tend to duplicate relatively frequently (Li et al. 2016); this is reflected in the small number of genes which were duplicated in the ingroup but not in the outgroup that are recovered in O’Toole et al. (2018). Davis and Petrov (2004) do not require that the genes examined are single copy in the outgroup, although it is mentioned that the observed effect holds if this restriction is used. Contrary to this, O’Toole et al. (2018) found that applying this restriction leaves no genes in the duplicable category at all. The large span of evolutionary time covered makes it difficult to find an appreciable number of genes which are duplicated in one lineage but not the other and further restricts the set of genes which can be used for the comparison of interest.

Here, we attempt to determine, in lineages other than primates, whether duplicable genes are ancestrally faster evolving or if duplication-induced redundancy accelerates evolutionary rates. We examine this in insect genomes, similarly to Davis and Petrov, but we remove other differences in study design by restricting the analysis to closely related species within the *Drosophila* lineage. We find that duplicable genes show higher ancestral rates of evolution when we use singleton orthologs in closely related outgroup species to estimate rates, and we do not find particular evidence to support the classical view of rate acceleration as a major trend following duplication though we note that rate acceleration is not mutually exclusive with our broader conclusions. The rate acceleration results may be affected by a subset of rapidly evolving genes being annotated as singletons when they may actually be genes which have simply suffered from homology detection failure (HDF) due to extensive sequence divergence.

## Results

### Faster Evolving Genes Are More Duplicable in the *Drosophila* Lineage

**Fig. 1.**
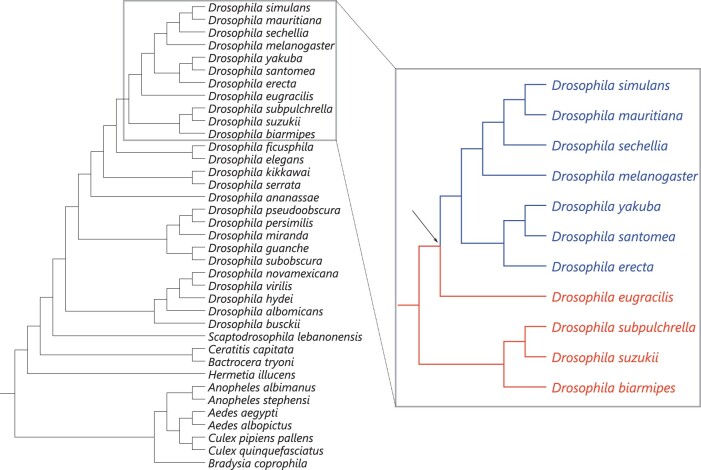
Project strategy. All species included in orthogroup inference and gene tree building are shown (left). The species considered in our analysis are shown in color with the outgroup for rate estimation shown below in red and the duplicability assessment group above in blue. The goal is to estimate a proxy for the evolutionary rate at the point indicated by the arrow which we infer predates the duplication event based on single-copy status in the outgroup.

**Fig. 2.**
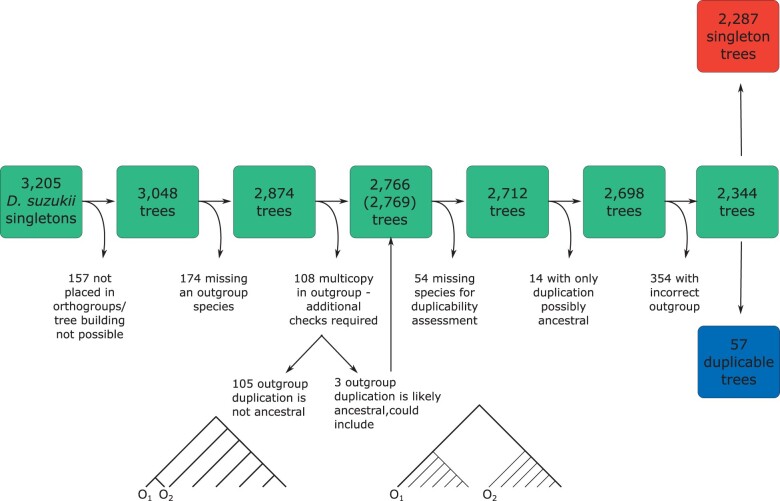
Quality filters on singleton and duplicable genes. It was required that singletons were present in an orthogroup and that a gene tree could be constructed from the orthogroup, and that the tree produced fulfilled a number of requirements. The numbers shown for the final data set are prior to rate calculation; additionally, we could not calculate evolutionary rates for 130 singleton trees and five duplicable trees. Final rate comparisons are thus between 2,157 singleton and 52 duplicable groups. O_1_ and O_2_ indicate duplicate copies in an outgroup species.

Following from the method in O’Toole et al. (2018), we selected a group of four *Drosophila* species as outgroup species to ascertain an ancestral single copy state and provide a proxy measure of the ancestral rate of evolution. A further seven species were designated as the group for duplicability assessment (fig. 1). We inferred gene trees for each ancestrally single-copy gene family, applied quality filters (fig. 2) and assessed duplicability, resulting in 2,157 singleton and 52 duplicable cases. This large difference in sample size between these groups is unsurprising as duplicable genes are expected to duplicate frequently and so our filtering for cases which are single copy in the outgroup species limits the number of these we can include. For each case, we calculated the proxy ancestral rate (between *Drosophila*  *suzukii* and *Drosophila*  *eugracilis*) as well as a rate measured in the clade of the duplication (between *D. suzukii* and *D. melanogaster*) to confirm the higher rate of evolution in duplicates.

**Fig. 3.**
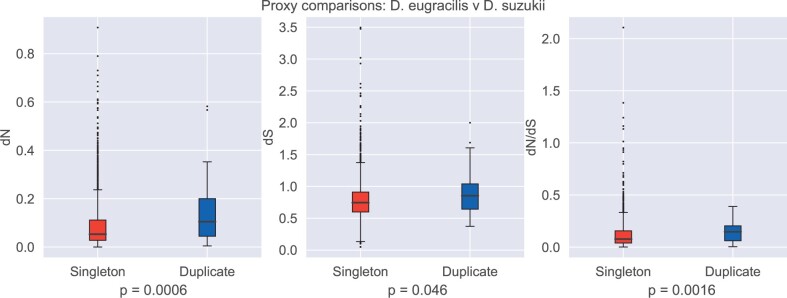
Duplicable genes are ancestrally faster evolving. Comparisons of the amount of nonsynonymous substitution (*d*_N_), synonymous substitution (*d*_S_), and the ratio between the two ($d_{N}/d_{S}$) for singleton and duplicable genes. Values were calculated in *Drosophila suzukii* and *Drosophila eugracilis* comparisons. *P* values are given for two-sided Mann–Whitney *U* tests and are Bonferroni corrected.

We find that genes that we have designated as duplicable show faster rates of evolution both within the clade where we assess duplicability (ingroup rates; see supplementary fig 3, Supplementary Material online) and in singleton outgroups, which we use as a proxy for the ancestral rate (fig. 3). The finding that the higher evolutionary rates predate any gene duplication event supports the conclusions of O’Toole et al. (2018) that biased duplicate retention in favor of ancestrally fast-evolving genes contributes to the observed differences in evolutionary rate between single-copy and duplicated genes.

### Potential Confounders Do Not Fully Explain the Faster Evolution Rate of Duplicable Genes

Having confirmed this core result, we next considered possible explanations for the higher rate of evolution in the set of duplicable genes. There are several gene features which are well known to correlate with rate of evolution, most notably expression level (Pál et al. 2001). Many of these features also show differences between groups of differing duplicability. We investigated whether any of these features explained the difference in evolutionary rate between duplicable and singleton genes.

**Fig. 4.**
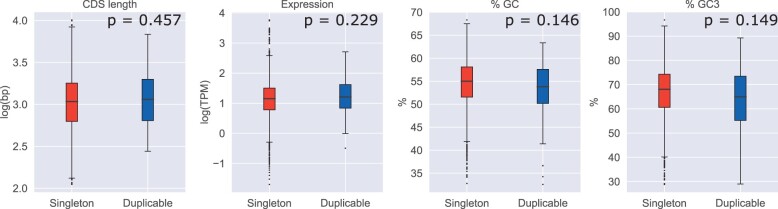
Confounding features show no significant difference between singleton and duplicable genes. *P* values given are for two-tailed Mann–Whitney *U* test and Bonferroni corrected.

**Fig. 5.**
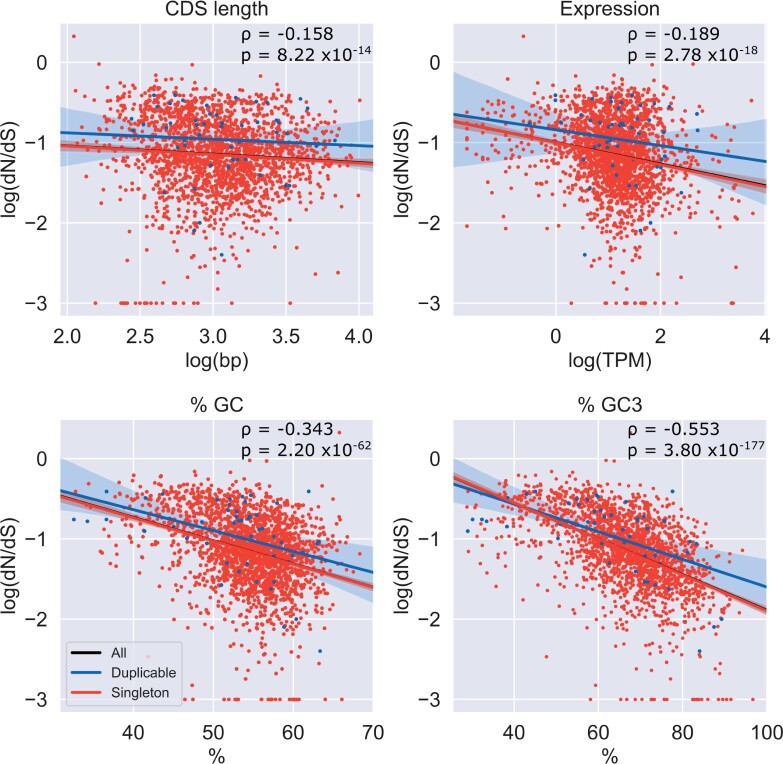
Ordinary least squares regression of rate on confounding features. *P* values and correlation coefficients are shown for Spearman’s correlation. Transformations are shown for each feature in axis labels.

For each of four features examined (CDS length, expression level, %GC content, and %GC3 content), we compared our singleton and duplicable groups and also calculated correlations between the feature and evolutionary rate. We do not find significant differences between the groups for any of the features considered (fig. 4), though we note duplicates show slightly higher values for CDS length and expression level and lower values for GC content. All also show significant negative correlations with evolutionary constraint (fig. 5). The GC content features, particularly GC3 content, show a relatively strong correlation (GC: ρ=−0.343, GC3:ρ=−0.553) in comparison to CDS length and expression level (CDS length: ρ=−0.158, expression:ρ=−0.189).

**Fig. 6.**
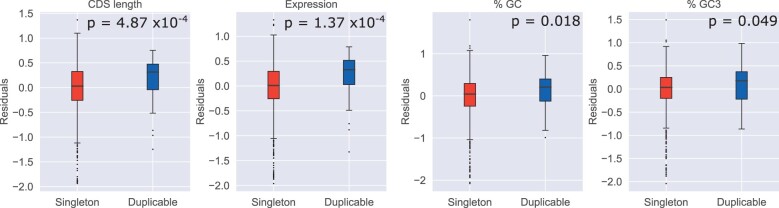
Residual comparisons show significant changes in *P* value. Comparisons are shown for residuals from regression rate on each feature. *P* values are shown for comparisons using a two-tailed Mann–Whitney *U* test. Change in *P* value relative to the original rate comparisons are all significant as determined by Monte–Carlo simulation with 100,000 iterations (CDS length: *P* = 0.012; expression, %GC, and %GC3: *P* < 0.00001).

Given these negative correlations, and the fact that O’Toole et al. (2018) had found such features to either mask or explain some of the difference in rates between these groups, we then regressed rate on each feature in order to control for any variation in rate explained by the feature in question and performed another comparison on the residuals (see Materials and Methods). In all cases, we find a significant change in *P* value for the residual comparison relative to the original rate comparison, suggesting that all features considered are in some way relevant to the observed difference in evolutionary rates. In the case of CDS length and expression level, we find that differences in these features may be masking the difference in rates, with slightly higher values for duplicable genes combined with negative correlations with rate giving an overall smaller difference than in the comparison controlling for these features (fig. 6). On the other hand, it seems that sequence composition may partially explain these differences in rates. Controlling for %GC content or %GC3 content increases the *P* value for the comparison, indicating that differences between singleton and duplicate groups are contributing to the rate difference (fig. 6). We additionally confirm these results are consistent when LOWESS regression is used (as in O’Toole et al. [2018]) rather than ordinary least squares (OLS) (see supplementary figs. 7 and 8, Supplementary Material online).

Our results for CDS length and GC content differ from those in primates, where CDS length was found to explain some rate difference and GC content was found to have no impact. In the case of CDS length, it is possible that misclassification of shorter, faster evolving duplicable genes as singletons has contributed to this difference (see later discussion of HDF). However, in the case of GC (and GC3) content, the difference may be legitimate. Species such as *Drosophila* with large population sizes are expected to show stronger effects of selection and in this case, we believe we are seeing evidence of more effective selection on codon usage in this lineage relative to primates. Preferred codons in *Drosophila*, both within our group of interest generally and in *D. suzukii* specifically, typically have high GC content and almost exclusively possess G/C in the third position (Vicario et al. 2007; Athey et al. 2017). Thus, this difference with primates may reflect selection on codon usage and may be explained by either more effective selection in *Drosophila* as compared with primates, or differences in codon preferences.

### Weak Evidence for Other Implications of Redundancy-Driven Rate Increase

A bias for retention of duplicates with faster evolving parent genes does not exclude the possibility that gene duplication also allows relaxation of constraint. We therefore additionally tested whether we could find evidence for other implications of the postduplication redundancy hypothesis, namely asymmetric rates of evolution and overall rate acceleration postduplication.

**Fig. 7.**
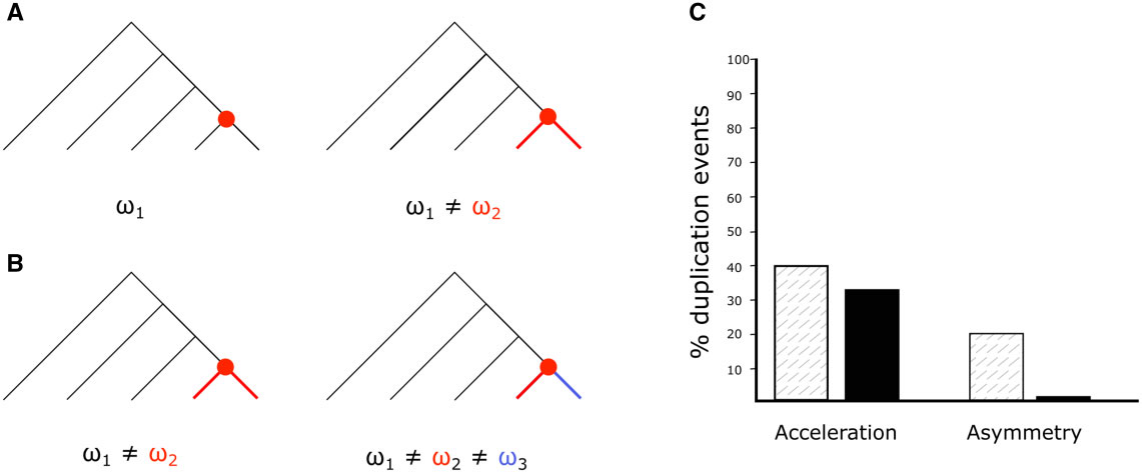
Comparison of differing rate models does not support rate acceleration or rate asymmetry postduplication. (*A*) Competing models in the test for rate acceleration. The null model assumes a constant background rate across the tree (ω_1_, indicated in black) whereas the alternative allows rate postduplication to vary (represented by the rate ω_2_ on red branches descending from the duplication node indicated by the red point). We find that in all cases of the second model performing better the postduplication rate is higher, and so we refer to these results as acceleration. (*B*) Competing models in the test for rate asymmetry. Null model assumes equal rates for both duplicate branches, the alternative allows differing rates (ω_2_ and ω_3_, represented in red and blue respectively). (*C*) Percentage of the 86 duplication events considered where the alternative model is found to be significantly more likely before multiple testing correction (hatched) and after (solid color).

Rate asymmetry in duplicates is supposed to originate from differences in functional constraint, with one copy assumed to maintain the functional role of the parent gene, whereas the other is free to evolve novel functions due to the redundancy. We investigated this idea and find little evidence for this difference in rate as a significant trend in our data set (fig. 7). When we estimate evolutionary rate using PAML under two models (either assuming the same rate for both branches postduplication or allowing these two rates to differ), we find that after multiple testing correction only about one out of 86 duplication events (1%) show a higher log likelihood under the model that allows for rate asymmetry, indicating that allowing for asymmetry does not produce a superior model. However, as multiple testing correction in this case is actually more generous to the hypothesis of no postduplication effect, we additionally use Fisher’s method for combining *P* values to consider all tests together. We find evidence here in favor of asymmetry ($\chi^{2}=292.96$; $P=2.48\times 10^{-8}$). Thus, although the evidence from this particular data set is not strong, one could argue that the conclusions depend on your prior expectations and the choice of null hypothesis. Previous reports of rate asymmetry (Wagner 2002; Conant and Wagner 2003), found the proportion of pairs evolving asymmetrically similar to that recovered here prior to multiple testing correction. The fact that we recover some support for asymmetry here in the large population size Drosophila, in contrast to O’Toole et al. (2018) working on lower population size primates, also is in agreement with the proposal by Wagner (2002) that the indirect effect on fitness could preclude detecting this effect in lineages with less efficient selection.

We additionally considered whether there was an overall increase in evolutionary rate postduplication for any of these genes. Again, we estimate rates under two models allowing for either one rate across the entire tree or for a differing rate postduplication (fig. 7). We find 67 out of 86 duplication events (78%) where the rate of evolution postduplication is estimated to be higher (a rate acceleration). However, in only 35 (40%) of these does the different rate produce a better model, and in only 28 of the cases (33%) is this model significantly better than the null after multiple testing correction. Similarly to the case of asymmetry, we additionally considered all duplicates as a group in order to be fair to the acceleration hypothesis and again find evidence in favor of this hypothesis ($\chi^{2}=702.30$; $P=3.43\times 10^{-65}$; Fisher’s method for combining *P* values). Nonetheless, we remain cautious in the interpretation of these results due to the possibility that the short time intervals interfere with the $d_{N}/d_{S}$ estimates (see Discussion). Additionally, it is important to consider statistical power. Individual hypothesis tests may lack the power to detect the difference in likelihood between models in less extreme cases, leading to the small quantity of cases we find to support the hypothesis of rate acceleration/asymmetry, with this issue largely solved by combining all tests. However, there is not much to suggest that likelihood ratio test used here is particularly likely to be underpowered (Irisarri and Zardoya 2013) and, if anything, such tests are more prone to false positives under certain circumstances (Aagaard and Phillips 2004).

### Fast-Evolving Singletons May Be Explained by Limitations of Orthology Inference

One curious observation is that, despite the overall faster rate of evolution of duplicable genes, there is a considerable number of fast-evolving singleton genes in our data set; of the top 5% of genes by rate all but four are classified as singletons. Although it is possible that this group could represent an interesting exception to faster evolving genes being more likely to duplicate, we first aimed to rule out technical explanations.

**Fig. 8.**
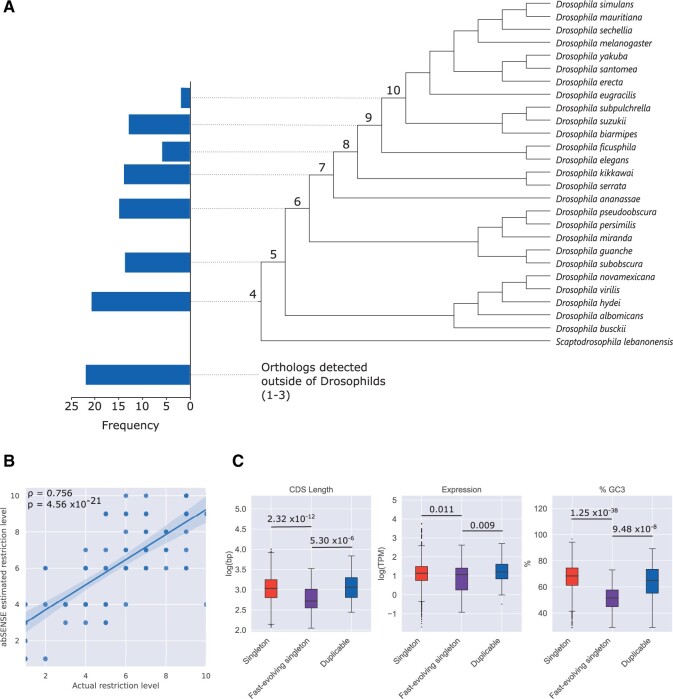
Fast-evolving singletons are a distinct group partially explained by HDF. (*A*) The distribution of singletons with orthologs identified at the node indicated but no further. (*B*) Correlation between observed restriction level (as indicated by numbering of nodes in [*A*]) and that predicted by abSENSE assuming no process other than constant sequence similarity decay for each fast-evolving singleton, Spearman’s correlation given. (*C*) Comparison between fast-evolving singletons and other groups in the analysis. *P* values are given for two-tailed Mann–Whitney *U* tests.

Such technical explanations of this group of fast-evolving singletons could include HDF in the case of especially fast-evolving genes. This is because fast evolution of some duplicated genes would result in extensive sequence divergence which may in turn lead to their misclassification as singletons due to the difficulty in detecting the paralogs (Wolfe 2004). Consistent with this possibility, a majority of fast-evolving singletons in our data set are taxonomically restricted (fig. 8), with 85 out of 107 (79%) lacking identified orthologs outside of the Drosophilids and 95 (89%) restricted to within fruit flies. Taxonomic restriction has been shown to be frequently explainable by HDF (Vakirlis et al. 2020; Weisman et al. 2020).

We investigated whether HDF was a plausible explanation for the restriction pattern we observe in these fast-evolving singletons. We used the software abSENSE (Weisman et al. 2020) to estimate at what point in the tree we would hypothetically lose the ability to detect orthologs even if there were orthologs present and the gene has been evolving at a constant rate. We find that HDF is a plausible explanation in 54% of cases (58/107), with abSENSE predicting loss of detection at or after the point where no orthologs are detected. We also observe a strong significant correlation between the observed taxonomic restriction level and that predicted by detection failure probabilities from abSENSE ($\rho =0.756,P=4.56\times 10^{-21}$; fig. 8*B*). Thus, these data support the idea that the fast-evolving singletons observed here may be misclassified due to missed paralogs.

We further considered whether our choice of tool for orthology inference contributed to the presence of cases with potentially missed paralogs and where in the process this failure may have occurred. Firstly, this pattern is not unique to our choice of orthology inference tool or settings, we find that other tools have similar, if not greater, levels of HDF. In the case of SonicParanoid, there are 114 genes which meet the criteria of being a fast-evolving singleton (97 in common with Orthofinder), while running Orthofinder with an ultrasensitive similarity search yielded 111 (102 in common with default parameter Orthofinder). Regardless of the settings used, we fail to recover any duplicable cases at all when OMA is used as the orthology inference tool (i.e., all genes are classified as singletons).

It seems that missed paralogs may involve failures at either the stage of homology detection, or clustering into orthogroups based on the similarity. We attempted to locate potential paralogs based solely on homology search rather than the final clustered groups and find very few cases, however, we note a slight increase in groups with possible missed paralogs when comparing default Orthofinder to Orthofinder ultrasensitive (4 cases as opposed to 2). Paralogs which could be detected at the stage of homology searches but which are not called as paralogs are potentially ancestral duplications that occurred prior to the divergence of our species set or could possibly have failed to cluster properly at the clustering step. As we recover very few of these cases, however, we believe the majority of errors would lie with HDF.

We examined whether the genes we designate as fast-evolving singletons are generally distinct from other groups in the data set and found that they are a distinct group in terms of all the confounding features we had earlier considered (fig. 8*C*). In all cases, these singletons show significantly lower values than either other singletons or duplicable genes, with the effect particularly pronounced for CDS length and GC3 content. As shorter, less conserved genes are more prone to HDF, and GC3 content is related to conservation level, this provides further support that HDF could be a plausible explanation for the faster evolutionary rates of these singletons

## Discussion

The work presented here extends previous observations on the faster rate of evolution of duplicable genes from primates to insects, and thus supports the interpretation that any apparent contradictions from previous studies (Davis and Petrov 2004; O’Toole et al. 2018) are not due to biological differences between the lineages (such as generation time and population size), and may instead reflect differences in the study design. Most notably, the choice of very distantly related outgroups may inadvertently create a bias for inclusion of slowly evolving genes because these are the ones where homology remains detectable for longer. The level of conservation in duplicated genes varies considerably with the age of duplication (Woods et al. 2013) and given that the initial data set is already predisposed to a higher level of conservation we might assume that the duplications detected may represent an older, more conserved subsample. Such ancient duplications may well be more conserved that singletons, however, these paralogs have long since moved beyond the stage of redundancy and initial duplicate evolution which we present here.

Prima facie our data lend support to the classical view that after duplication there is also rate acceleration, albeit not typically associated with much rate asymmetry. It is thus possible that both fast-evolving genes duplicate more and that after duplication genes can accelerate in their evolution. However, there may be reason to be cautious about the evidence for rate acceleration in this data set. In all cases where there is a change, the rate of evolution (approximated by $d_{N}/d_{S}$) after duplication is higher than that before duplication. Although consistent with extremely common rate acceleration, there may be alternative explanations. Indeed, it has previously been argued that unresolved polymorphism between recently diverged species can lead to false $d_{N}/d_{S}$ signals (Rocha et al. 2006). This is owing to a time lag for a weakly deleterious mutation to be removed from a population. Indeed, if one captures mutations at the point at which they occur (e.g., comparing parents and offspring) $d_{N}/d_{S}$ should be unity (or near unity if we allow for rare lethal mutations) as the nonsynonymous mutations have yet to be removed from the population. As Rocha et al. (2006) observe, the expectation is that as the distance between two comparators increases so $d_{N}/d_{S}$ declines. For the Drosophila species, the postduplication branch lengths are small hence there remains the possibility that apparent rate acceleration is an artifact of short branch lengths and unresolved polymorphisms contaminating pairwise analyses. That polymorphism levels are higher in flies than in primates adds to the concern. Masking of polymorphic sites in all genes in all species could enable a possible test of this alternative explanation but is currently not possible.

Additionally, some of our results may be impacted by limitations in homology detection and orthology inference. If particularly quickly evolving duplicable genes are misclassified as singletons this would mean that the association between evolutionary rate and duplicability is underreported here. By contrast, the impact of such HDF on the estimation of postduplication rate asymmetry is harder to predict. Asymmetrically evolving duplicates are known to be difficult to place correctly in orthology inference as the faster evolving paralog can have a much greater sequence distance to other orthologs than its sister and be incorrectly clustered outside of the group as an out-paralog (Train et al. 2017). As such, any missed duplication events may be biased toward cases where rate asymmetry has occurred, leading to a potential underdetection of rate asymmetry here.

It is not clear why reliable duplicate detection appears to be more difficult in *Drosophila* than in primates. There is known to be quick turnover and fast-acting selection on new duplicates in *Drosophila* (Jiang and Assis 2017; Li et al. 2019), possibly as a result of more efficient selection in a high *N*_e_ species. Potentially this could drive faster loss or divergence of paralogs in this lineage, thus making them more difficult to detect even over relatively short stretches of time. We do not address here the possibility that some of the fast-evolving singletons may be legitimate de novo genes (Vakirlis et al. 2020). Our tests with abSENSE do not rule this out as a possibility, only confirm that it is possible the taxonomic restriction pattern has arisen through HDF.

In this work, we have resolved previous conflicts in results regarding the source of faster evolutionary rates of duplicable genes. We show that a faster rate in duplicable genes prior to duplications is not limited to the primate lineage and this finding is likely broadly applicable. Overall, our observations support the idea that at least some of the differences in rate between singleton and duplicated genes can be traced to before the duplication event occurs and that biases in duplicability should be included as a potentially significant factor in any explanation of these rate differences. The nature of the biases is not clear. Previous work in *C. elegans* supports the interpretation of duplication as just another kind of variation (Woods et al. 2013), and genes that are less constrained in sequence evolution are also generally less constrained in terms of copy number evolution. However, it is interesting to speculate whether the faster rate of evolution might contribute to a greater chance of duplicate fixation through functional diversification.

## Materials and Methods

### Inference of Orthologies

Proteomes for 37 good quality (contig N50 ≥200 kb and scaffold N50 ≥500 kb) *Diptera* genomes were downloaded via the NCBI FTP site (see supplementary table 1, Supplementary Material online). Protein sequences were used with a number of orthology inference tools to infer orthologous relationships and groups. The main body of analysis was based on orthologous relationships from Orthofinder with default settings (Emms and Kelly 2019), with additional checks for duplicate detection capacity using Orthofinder with the ultrasensitive setting, SonicParanoid (Cosentino and Iwasaki 2019), and OMA (Train et al. 2017), which was run three times varying the “InParalogTol” parameter.

### Selection of Singleton Groups and Tree Building

Candidate singletons were defined for *D. suzukii* as genes with no nonself BLAST hit with E≤0.1 when searching the genome against itself, and orthogroups were then extracted for this set. Each orthogroup was aligned using MUSCLE (Edgar 2004) and trees were built from these alignments using IQ-TREE with models being selected from WAG, LG, and JTT (Kalyaanamoorthy et al. 2017; Hoang et al. 2018; Minh et al. 2020). Trees were processed using the ete3 python package (Huerta-Cepas et al. 2016). The strict initial selection of singletons provides confidence in singleton status in the outgroup species, though it is possible that genes with very ancient duplications that predate our time-frame of interest are excluded at this step. We note that use of a more relaxed cutoff for singletons (genes with no nonself BLAST hit with $E\leq 1\times 10^{-4}$) does not meaningfully affect our final results (see supplementary figs. 1 and 2, Supplementary Material online) and so we expect that these results are robust to any such effect.

### Data Set Filtering and Evolutionary Rate Calculation

A number of filtering steps were carried out to ensure the trees used in the final comparisons did not violate any of our assumptions (fig. 2).

Sequence evolution parameters (*d*_N_, *d*_S_, and $d_{N}/d_{S}$) were calculated using the codeml module of PAML (Yang 2007) with model = −2 for pairwise rate calculation and all other parameters set to defaults. Cases where *d*_S_ exceeded 4 were excluded as synonymous substitutions were considered too saturated at this point to give reliable estimates, although we do find significant differences between duplicable and singleton groups even in the absence of this filter (supplementary figs. 4 and 5, Supplementary Material online). For ingroups, pairwise rates were calculated between *D. melanogaster* and *D. eugracilis*, whereas for proxy ancestral rates, pairwise rates were calculated between *D. suzukii* and *D. eugracilis*.

### PAML Branch Models

To test different hypotheses regarding rate changes after gene duplication, we estimated evolutionary rates under a number of different models for each duplication event in the gene trees for our duplicable gene set. Duplication events were determined using ete3 and for each case, we estimated rates under three models (see fig. 7). Firstly, the case where all rates are assumed equal (model = 0); secondly, the case where the rate is allowed to differ postduplication but rates on both branches are assumed equal (model = 2 with all branches descending from the duplication labeled with the same rate); thirdly, the case where rates were allowed to differ on the two branches (model = 2 with branches descending from the duplication event labeled with two different rates).

For each model, the number of parameters and the log likelihood were extracted and we performed a likelihood ratio test ($\chi =2(\text{Ln}L_{\text{max}}-\text{Ln}L_{\text{min}})$) to determine if a) allowing the rate to differ from the rest of the tree postduplication produces a better model than a model with all rates assumed equal; and b) allowing the duplicates to differ in rate produces a better model than assuming both duplicates share the same rate. The resulting *P* values were corrected using FDR.

### Quantification of Potential Confounders

For each gene under consideration, we determined values for CDS length, % GC content, % GC3 content based on the longest CDS. Expression values were estimated in transcripts per million using RSEM v 1.3.3 (Li and Dewey 2011). RNA-seq data for *D. suzukii* were obtained from SRA (SRR1002988 and SRR100289), trimmed using Trim Galore (Martin 2011) and aligned with the STAR aligner v 2.7.7a (Dobin et al. 2013) using default parameters.

### Assessment of Confounder Contribution

In order to assess whether any of our confounding features could be contributing to the observed difference in rate between the singleton and duplicable groups, we endeavored to compare rate between these groups with influence from a given confounder removed. We achieved this by regressing rate on each feature in turn to obtain residuals which we took as a measure of rate independent of any variance explained by the feature under consideration. We then compared these residuals between singletons and duplicable genes using a Mann–Whitney *U* test, taking a reduction in *P* value compared with the original rate comparison to indicate the feature was masking a difference in rate (i.e., removing the influence of the feature had made the groups less similar). By the same logic, an increase in *P* value was taken to indicate the feature contributes to the observed difference in raw rates.

The regression models to determine residuals for each feature were based on OLS regression, that is, a linear model. We investigated that our data met the assumptions required for such models and find no meaningful deviations save for a minor negative skew in the residual distribution following log transformation of rate and, in the case of CDS length and expression, of the predictor (see supplementary fig. 6, Supplementary Material online). As we were considering our results in light of those presented in O’Toole et al. (2018), we additionally performed the same analysis with LOWESS regression as used in that work. LOWESS regression is a smoothing approach that uses local data structure to fit a curve to a data set. We favor OLS as the local nature of LOWESS makes these models less reliable at values where data are not densely sampled, however, we include the LOWESS results to show that the choice of regression approach does not affect our conclusions.

We determined whether the change in *P* value between the raw rate comparison and the residual comparison was statistically significant using Monte–Carlo simulation. For each feature, we randomly permuted the values and regressed rate on these values 100,000 times in order to create a distribution for the residual comparison between singleton and duplicable genes. Based on this, we estimate the *P* value for the change in *P* value as the probability of finding a higher value in the case of an increase and the probability of finding a lower value in the case of a decrease. We expect that this approach to judging the significance of the change in *P* value should account for any effects nonspecific to the contribution of the feature in question, such as a decrease in variance that we would expect to observe in the residuals compared with raw rates.

Previous work examining the determinants of evolutionary rate has shown standard regression methods perform poorly in the presence of noisy, correlated predictors, as noisy predictors can only be imperfectly controlled for given the imprecise measurements, and suggested principle components analysis (PCA) regression was superior (Drummond et al. 2006). We chose not the implement PCA regression in this case as, firstly, we were not considering our potential confounders together in a single model so multicollinearity is not an issue within each model and, secondly, we were not necessarily interested in assessing the relative contributions of each confounder to explaining rate variation. Rather, we sought to investigate whether any given confounder was related specifically to the difference in rates observed between singleton and duplicable groups. In the case of our method, failure to fully control for a confounder due to noise in measurements should only serve to minimize the change in *P* value we observe, as some variation due to the confounder has not been accounted for in the residuals. Thus, if this issue exists for any of our measurements we expect it would not lead to spurious significant results.

### Reassessment of Duplication Status for Fast-Evolving Singletons

The fast-evolving singletons group was operationally defined as singletons in the top 5% of the final set of genes by evolutionary rate. The level of taxonomic restriction was estimated as the most distant node from the focal clade where orthologs were detected. We used the abSENSE software with *D. melanogaster* as the focal species to confirm that these restriction levels could plausibly be explained by HDF, using the software as described in Weisman et al. (2020) with the difference that insect BUSCO genes were used for distance estimation between species to increase the number of orthologs recovered. Orthologs included were those defined by Orthofinder. For the bitscores required, we ran DIAMOND (Buchfink et al. 2021) searches for each pair of species with a generous E-value cutoff of 10 in order to maximize the ortholog pairs with a recovered bitscore. In cases where the ortholog pair did not produce a hit in the DIAMOND output, the ortholog was treated as undetected. For this analysis, the restriction level was defined as the most distant node where at least one species has 50% or higher probability of detection at E≤0.0001.

In order to assess whether we could detect paralogous copies for these assumed singletons, we also looked at all detected homologies from the similarity search step for Orthofinder (both default and ultrasensitive searches).

### Additional Statistical Methods

Pairwise comparisons were carried out using the Mann–Whitney *U* test with Bonferroni correction for multiple testing where appropriate. Regression models for examining the effect of possible confounders were constructed using the statsmodels Python module (Seabold and Perktold 2010). Residuals from regressing rate on each confounder were compared between duplicate groups and any change in *P* value relative to the original comparison was assessed for significance by Monte–Carlo simulation with *n* = 100,000.

## Supplementary Material

Supplementary data are available at *Genome Biology and Evolution* online.

## Acknowledgments

This work is supported by funding from the European Research Council Grant Agreements 309834 and 771419 to A.McL.

## Data Availability

Genomic resources used in this text are available as given in supplementary table 1, Supplementary Material online. Other data and analysis code are available on Github at https://github.com/ZoeVance/drosophilaDuplicability (last acccessed January 12, 2022).

## Supplementary Material

evac003_Supplementary_Data
