# Supplementary material for: Evidence from Drosophila Supports Higher Duplicability of Faster Evolving Genes
Source: Genome Biol Evol. 2022 Jan 9;14(1):evac003. doi: 10.1093/gbe/evac003 (PMC8765793; doi:10.1093/gbe/evac003)
Supplement: evac003_Supplementary_Data [file evac003_supplementary_data.zip › Vance_SuppInfo.pdf]

Evidence from Drosophila supports higher duplicability of faster evolving genes - Supplementary Information

Zoe Vance[1], Lukasz Niezabitowski[1], Laurence D. Hurst[2], and Aoife McLysaght[1,3]

[1]*Smurfit Institute of Genetics, Trinity College Dublin, Dublin 2, Ireland*
[2]*Department of Biology and Biochemistry, University of Bath, Bath, Somerset, UK BA2 7AY*
[3]*Correspondence to aoife.mclysaght@tcd.ie*

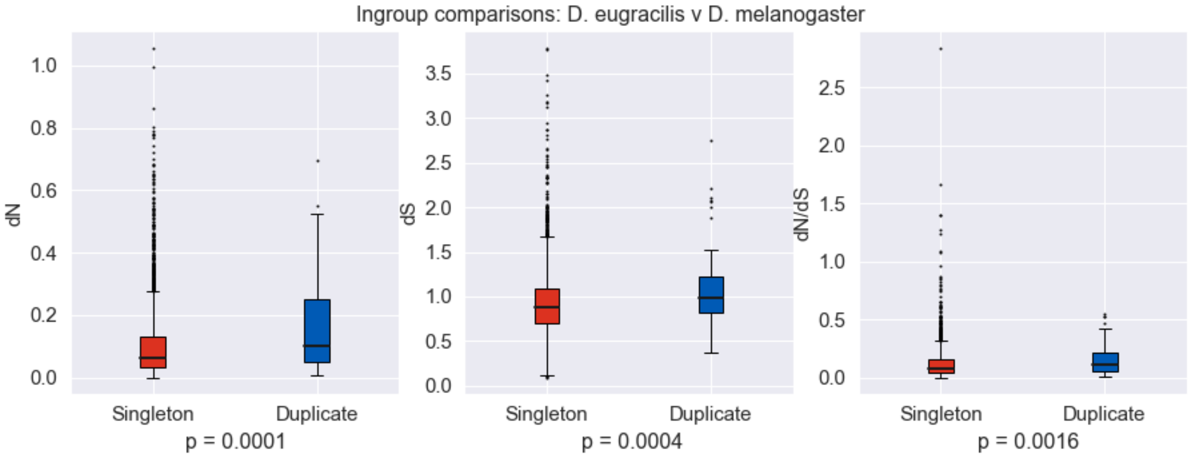

Supplementary Figure 1: Ingroup rate comparisons in duplicability assessment clade with more relaxed starting singleton definition. We confirmed the known trend that duplicated genes are faster evolving by comparing rates within the set of species where we assessed duplicability. A d_S threshold of 4 is applied and starting set of singletons was defined with an E-value cutoff of 10^{-4}. P-values are given for two-sided Mann-Whitney U-tests.

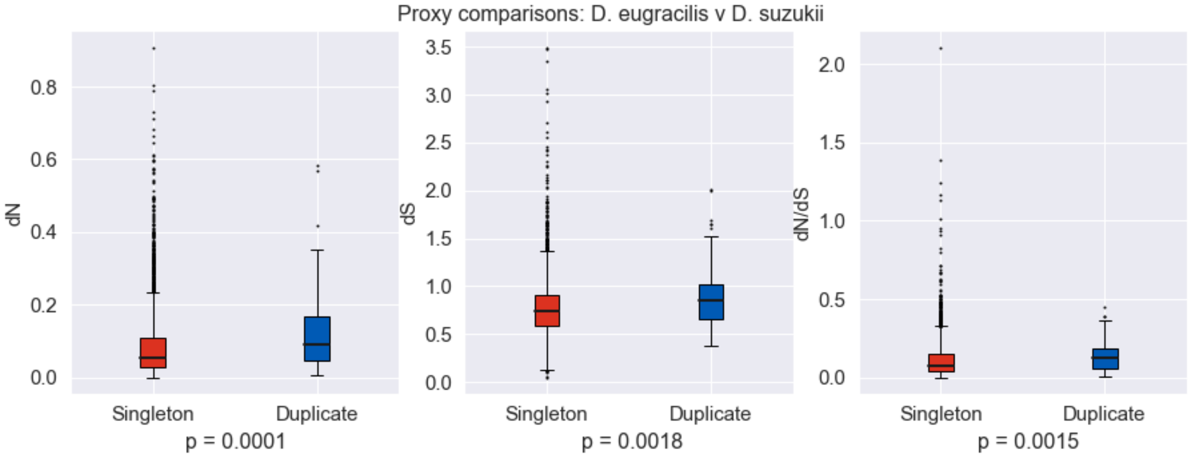

Supplementary Figure 2: Proxy ancestral rate comparisons with more relaxed starting singleton definition. Proxy ancestral evolutionary rates are faster in duplicable groups when using a more relaxed set of singletons as the initial dataset. A d_S threshold of 4 is applied and starting set of singletons was defined with an E-value cutoff of 10^{-4}. P-values are given for two-sided Mann-Whitney U-tests.

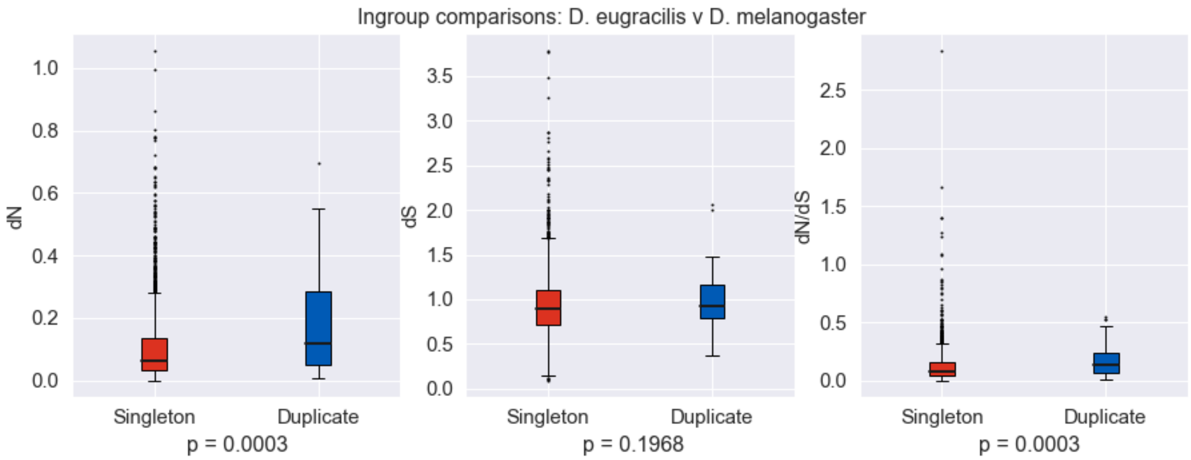

Supplementary Figure 3: Duplicated genes are faster evolving. We confirmed the known trend that duplicated genes are faster evolving by comparing rates within the set of species where we assessed duplicability. P-values are given for two-sided Mann-Whitney U-tests.

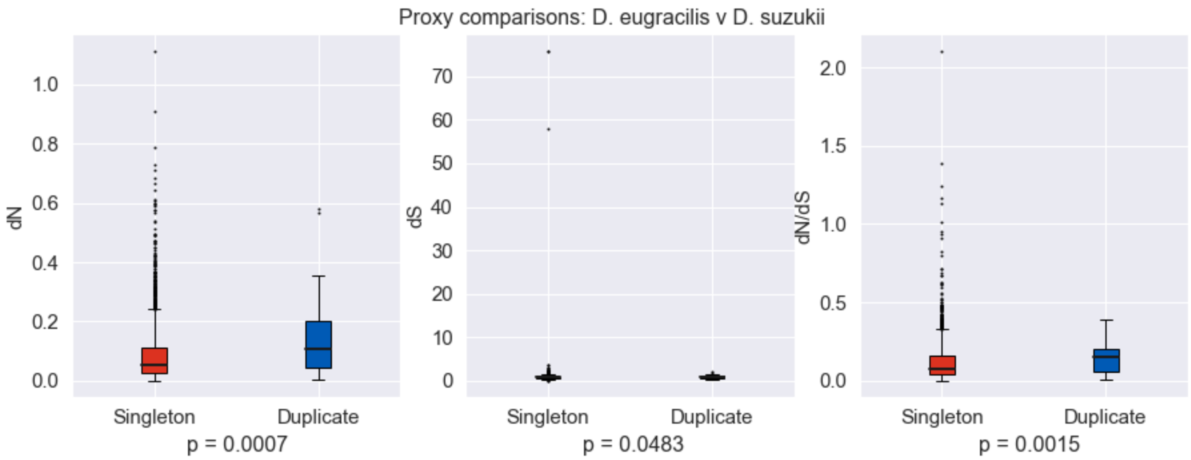

Supplementary Figure 4: Proxy ancestral rate comparisons with no d_S filter applied. Proxy ancestral evolutionary rates differ between singleton and duplicable genes before the removal of cases where the rate of synonymous substitution is saturated. P-values are given for two-sided Mann-Whitney U-tests.

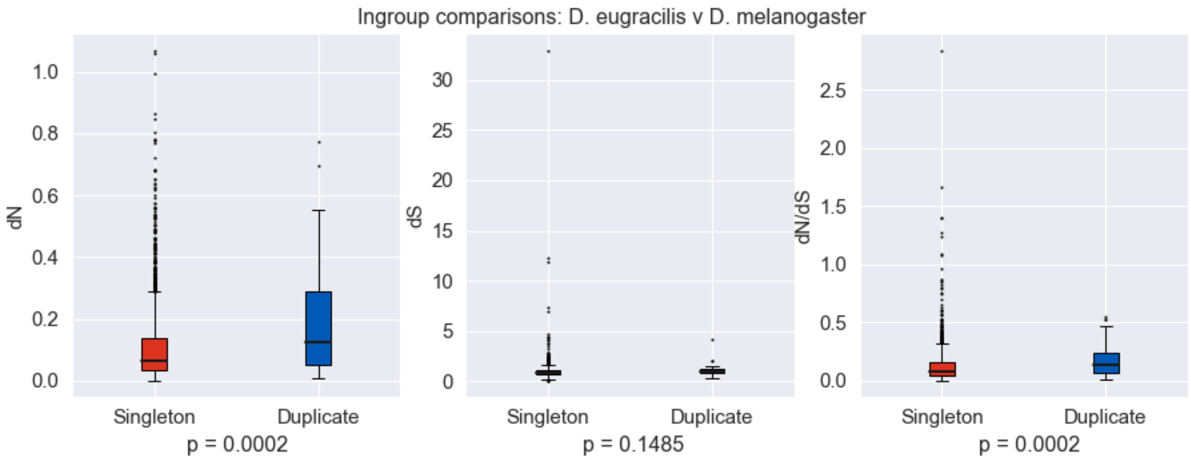

Supplementary Figure 5: Ingroup rate comparisons with no *dS* filter applied. Evolutionary rates as measured within the duplicability assessement clade differ between singleton and duplicable genes before the removal of cases where the rate of synonymous substitution is saturated. P-values are given for two-sided Mann-Whitney U-tests.

Species	NCBI FTP location
DALB	GCF/009/650/485/GCF_009650485.1_drosAlbom151121751.03v1
DANA	GCF/003/285/975/GCF_003285975.2_DanaRS2.1
DBIA	GCF/000/233/415/GCF_000233415.1_Dbia_2.0
DBUS	GCF/011/750/605/GCF_011750605.1_ASM1175060v1
DELE	GCF/000/224/195/GCF_000224195.1_Dele_2.0
DERE	GCF/003/286/155/GCF_003286155.1_DereRS2
DEUG	GCF/000/236/325/GCF_000236325.1_Deug_2.0
DFIC	GCF/000/220/665/GCF_000220665.1_Dfic_2.0
DGUA	GCF/900/245/975/GCF_900245975.1_DGUA_6
DHYD	GCF/003/285/905/GCF_003285905.1_DhydRS2
DKIK	GCF/000/224/215/GCF_000224215.1_Dkik_2.0
DMAU	GCF/004/382/145/GCF_004382145.1_ASM438214v1
DMIR	GCF/003/369/915/GCF_003369915.1_D.miranda_PacBio2.1
DMEL	GCF/000/001/215/GCF_000001215.4_Release_6_plus_ISO1_MT
DNOV	GCF/003/285/875/GCF_003285875.2_DnovRS2.1
DPER	GCF/003/286/085/GCF_003286085.1_DperRS2
DPSE	GCF/009/870/125/GCF_009870125.1_UCI_Dpse_MV25
DSAN	GCF/016/746/245/GCF_016746245.1_Prin_Dsan_1.0
DSEC	GCF/004/382/195/GCF_004382195.1_ASM438219v1
DSER	GCF/002/093/755/GCF_002093755.1_Dser1.0
DSIM	GCF/016/746/395/GCF_016746395.1_Prin_Dsim_3.0
DSUB	GCF/008/121/235/GCF_008121235.1_UCBerk_Dsub_1.0
DSUP	GCF/014/743/375/GCF_014743375.2_RU_Dsub_v1.1
DSUZ	GCF/013/340/165/GCF_013340165.1_LBDM_Dsuz_2.1.pri
DVIR	GCF/003/285/735/GCF_003285735.1_DvirRS2
DYAK	GCF/016/746/365/GCF_016746365.1_Prin_Dyak_Tai18E2_2.0
AAEG	GCF/002/204/515/GCF_002204515.2_AaegL5.0
AALB	GCF/013/758/885/GCF_013758885.1_VT_AalbS3_pri_1.0
AEAL	GCF/006/496/715/GCF_006496715.1_Aalbo_primary.1
ASTE	GCF/013/141/755/GCF_013141755.1_UCI_ANSTEP_V1.0
BCOP	GCF/014/529/535/GCF_014529535.1_BU_Bcop_v1
BTRY	GCF/016/617/805/GCF_016617805.1_CSIRO_BtryS06_freeze2
CCAP	GCA/905/071/925/GCA_905071925.1_EGII-3.2.1
CPIP	GCF/016/801/865/GCF_016801865.1_TS_Cpip_V1
CQUI	GCF/015/732/765/GCF_015732765.1_VPISU_Cqui_1.0_pri_pater
HILL	GCF/905/115/235/GCF_905115235.1_iHerIll2.2.curated.20191
SLEB	GCF/003/285/725/GCF_003285725.1_SlebRS2

Supplementary Table 1: Data location for species used. Locations are given for data directories in the NCBI FTP site (ftp.ncbi.nlm.nih.gov) where CDS sequences and associated translations were obtained. Full species names for the abbreviations in this table are given in Supplementary Table 2.

**Abbreviation**	**Species**
DALB	*Drosophila albomicans*
DANA	*Drosophila ananassae*
DBIA	*Drosophila biarmipes*
DBUS	*Drosophila busckii*
DELE	*Drosophila elegans*
DERE	*Drosophila erecta*
DEUG	*Drosophila eugracilis*
DFIC	*Drosophila ficusphila*
DGUA	*Drosophila guanche*
DHYD	*Drosophila hydei*
DKIK	*Drosophila kikkawai*
DMAU	*Drosophila mauritiana*
DMIR	*Drosophila miranda*
DMEL	*Drosophila melanogaster*
DNOV	*Drosophila novamexicana*
DPER	*Drosophila persimilis*
DPSE	*Drosophila pseudoobscura*
DSAN	*Drosophila santomea*
DSEC	*Drosophila sechellia*
DSER	*Drosophila serrata*
DSIM	*Drosophila simulans*
DSUB	*Drosophila subobscura*
DSUP	*Drosophila subpulchrella*
DSUZ	*Drosophila suzukii*
DVIR	*Drosophila virilis*
DYAK	*Drosophila yakuba*
AAEG	*Aedes aegypti*
AALB	*Anopheles albimanus*
AEAL	*Aedes albopictus*
ASTE	*Anopheles stephensi*
BCOP	*Bradysia coprophila*
BODO	*Bradysia odoriphaga*
BTRY	*Bactrocera tryoni*
CCAP	*Ceratitis capitata*
CPIP	*Culex pipiens pallens*
CQUI	*Culex quinquefasciatus*
HILL	*Hermetia illucens*
SLEB	*Scaptodrosophila lebanonensis*

Supplementary Table 2: Abbreviations used. Full species names for abbreviations used in data processing and in Supplementary Table 1.

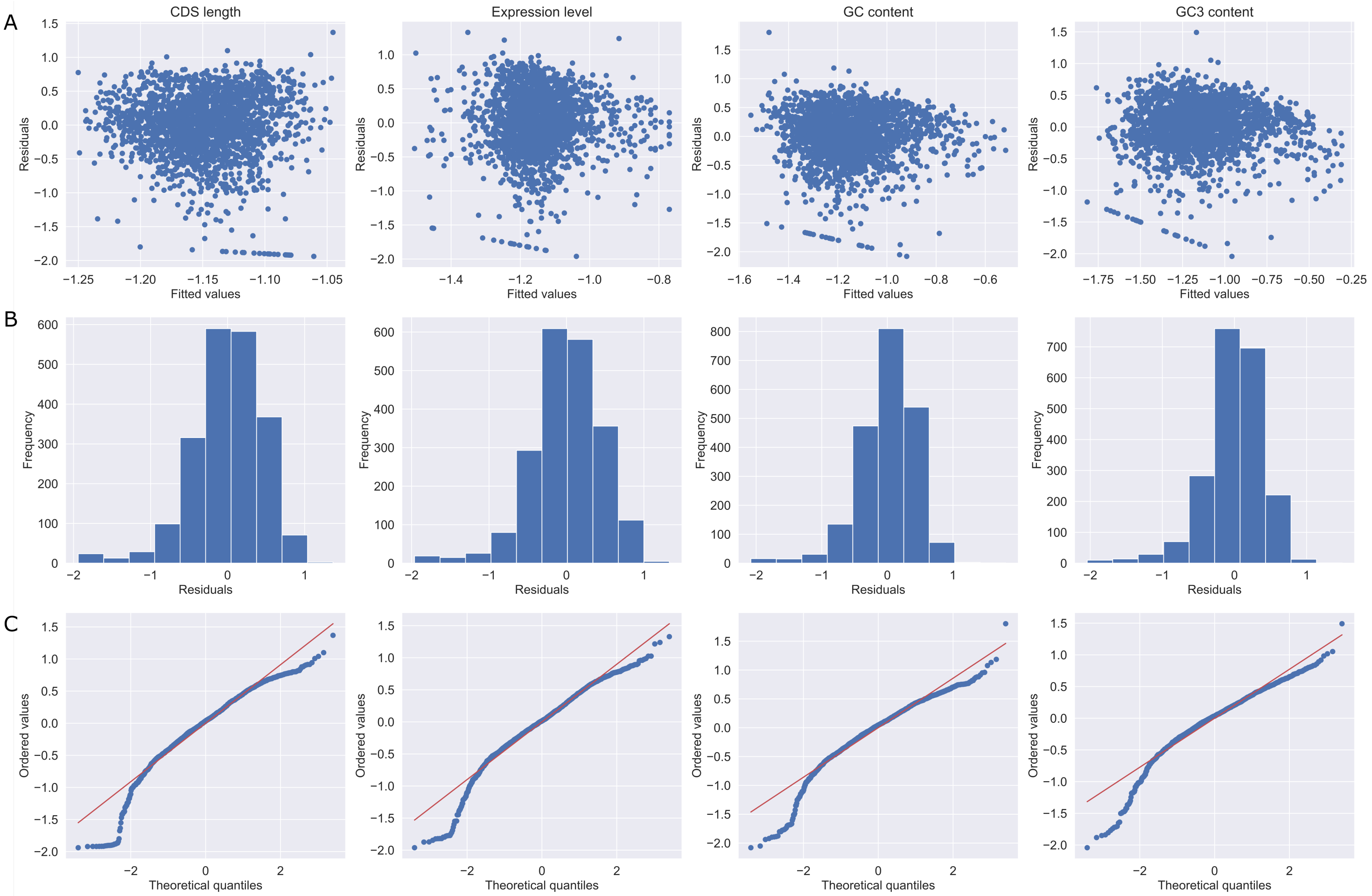

Supplementary Figure 6: Assessment of OLS regression assumptions. (A) Residuals for regression models for all confounders considered vs. fitted value. We do not observe substantial heteroskedasticity or other patterns of concern. (B) Distribution of residuals, residuals are approximately normally distributed around 0. (C) Q-Q plot of residuals vs. theoretical normal distribution indicates a slight negative skew.

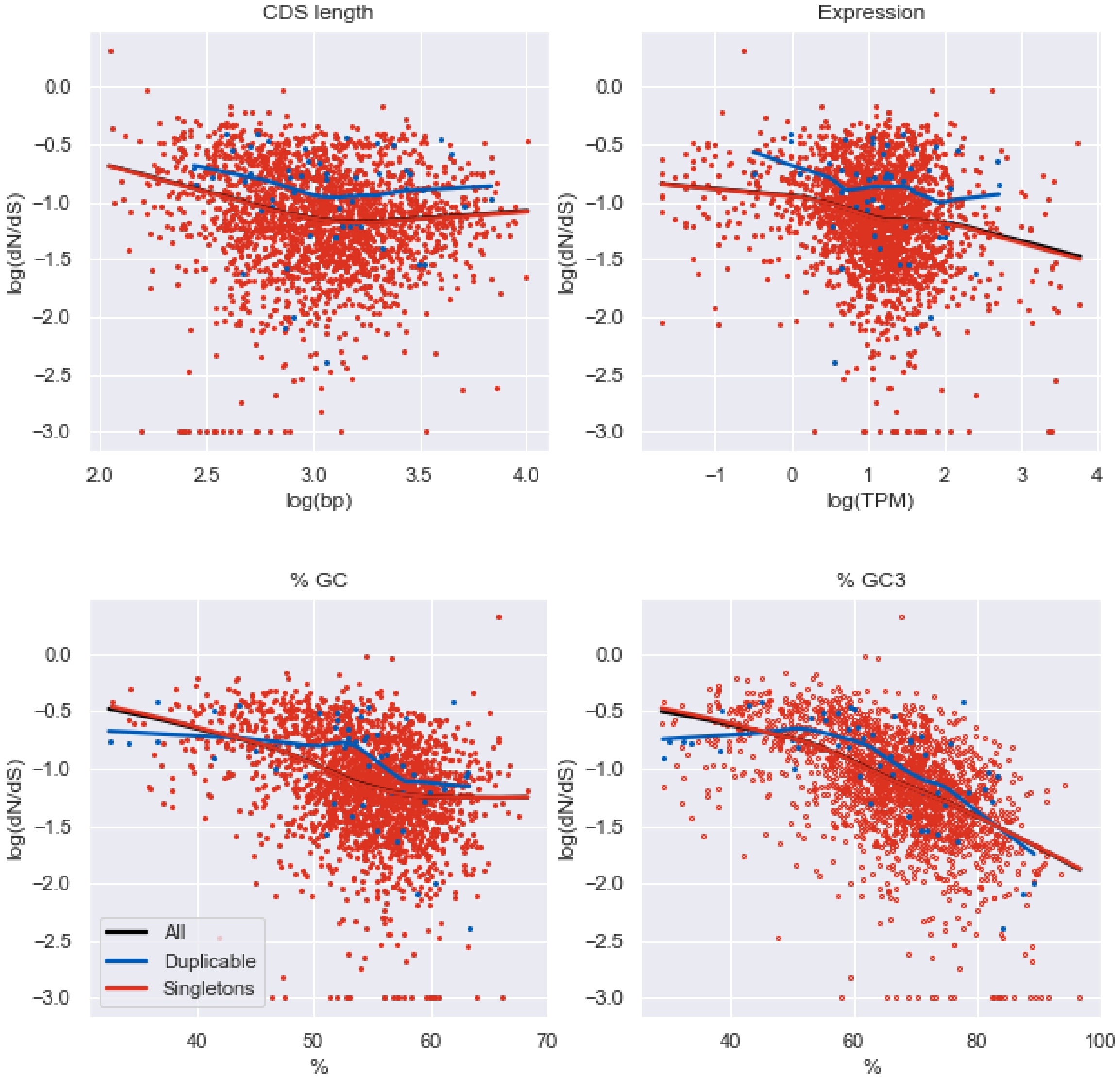

Supplementary Figure 7: LOWESS regression on confounders. Evolutionary rate was regressed on each of the features shown using LOWESS with f=3.

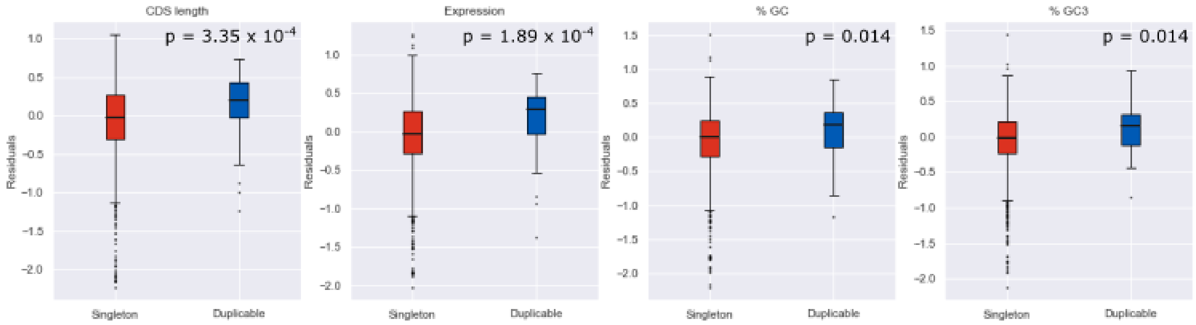

Supplementary Figure 8: Comparison of LOWESS residuals from regressing rate on counfouders. P-values are given for two-tailed Mann-Whitney U tests.
